# Verification of In Vitro Anticancer Activity and Bioactive Compounds in Cordyceps Militaris-Infused Sweet Potato Shochu Spirits

**DOI:** 10.3390/molecules29092119

**Published:** 2024-05-03

**Authors:** Kozue Sakao, Cho Sho, Takeshi Miyata, Kensaku Takara, Rio Oda, De-Xing Hou

**Affiliations:** 1 The United Graduate School of Agricultural Sciences, Kagoshima University, 1-21-24 Korimoto, Kagoshima 890-0065, Japan; sakaok24@agri.kagoshima-u.ac.jp (K.S.); miyata@agri.kagoshima-u.ac.jp (T.M.); k-takara@agr.u-ryukyu.ac.jp (K.T.); 2 Graduate School of Agriculture, Forestry and Fisheries, Kagoshima University, Kagoshima 890-0065, Japan; 3 Kirishima Shuzo Co., Ltd., 4-28-1 Shimokawahigashi, Miyakonojo, Miyazaki 885-8588, Japan; zhang_chau@kirishima.co.jp; 4 Faculty of Agriculture, University of the Ryukyus, 1 Senbaru, Nishihara, Okinawa 903-0213, Japan

**Keywords:** cordyceps militaris, Shochu spirits, adenosine derivatives, cordycepin, N^6^-(2-hydroxyethyl)-adenosine, anticancer activity, A3 adenosine receptor

## Abstract

Many liqueurs, including spirits infused with botanicals, are crafted not only for their taste and flavor but also for potential medicinal benefits. However, the scientific evidence supporting their medicinal effects remains limited. This study aims to verify in vitro anticancer activity and bioactive compounds in shochu spirits infused with Cordyceps militaris, a Chinese medicine. The results revealed that a bioactive fraction was eluted from the spirit extract with 40% ethanol. The infusion time impacted the inhibitory effect of the spirit extract on the proliferation of colon cancer-derived cell line HCT-116 cells, and a 21-day infusion showed the strongest inhibitory effect. Furthermore, the spirit extract was separated into four fractions, A-D, by high-performance liquid chromatography (HPLC), and Fractions B, C, and D, but not A, exerted the effects of proliferation inhibition and apoptotic induction of HCT-116 cells and HL-60 cells. Furthermore, Fractions B, C, and D were, respectively, identified as adenosine, cordycepin, and N^6^-(2-hydroxyethyl)-adenosine (HEA) by comprehensive chemical analyses, including proton nuclear magnetic resonance (^1^H-NMR), Fourier transform infrared spectroscopy (FT-IR), and electrospray ionization mass spectrometry (ESI-MS). To better understand the bioactivity mechanisms of cordycepin and HEA, the agonist and antagonist tests of the A3 adenosine receptor (A3AR) were performed. Cell viability was suppressed by cordycepin, and HEA was restored by the A3AR antagonist MR1523, suggesting that cordycepin and HEA possibly acted as agonists to activate A3ARs to inhibit cell proliferation. Molecular docking simulations revealed that both adenosine and cordycepin bound to the same pocket site of A3ARs, while HEA exhibited a different binding pattern, supporting a possible explanation for the difference in their bioactivity. Taken together, the present study demonstrated that cordycepin and HEA were major bioactive ingredients in Cordyceps militaries-infused sweet potato shochu spirits, which contributed to the in vitro anticancer activity.

## 1. Introduction

Liqueurs, including botanically infused spirits, are traditional alcoholic beverages that have been consumed for many years. These plants have a variety of flavors, aromas, and sometimes healthy properties. Herbal and fruit liqueurs are considered spirits with functional properties due to the presence of bioactive extractable compounds in their ingredients [1,2]. Plum liqueurs have been made for a long time in Asian countries, including Japan, by infusing Ume fruit in alcohol [3]. Nowadays, homemade soaked liquors are made from various fruits, herbs, and green and black teas. Although they are expected to contribute to health, there is very little research on the functional properties of the ingredients used in fruit liqueurs and spirits.

Sweet potato shochu is a distilled spirit that was introduced to Japan in the 14th and 15th centuries. Sweet potato shochu is rich in longer-chain alcohols, esters, monoterpene alcohols, and organic acid. Sweet potato shochu is also known for its low alcohol and calorie contents and is sugar-free This feature makes it as a popular choice for people who are conscious of preventing lifestyle-related diseases. Moreover, sweet potato shochu has been reported to have a functional effect on thrombus dissolution [4].

Cordyceps sinensis is a type of mushroom that has been used for more than 3000 years and is mentioned in the oldest Chinese pharmacopeia as a traditional herbal medicine [5]. Of these, Cordyceps militaris is a representative species that has been traditionally used in Chinese medicine and as a medicinal food. Cordyceps militaris is a fungus that contains various bioactive components, including adenosine, cordycepin, Cordicepic acid (D-mannitol), nucleic acid, polyphenols, and oligosaccharides [6,7]. Studies have shown that it possesses antitumor, immunostimulatory, anti-inflammatory, and hypoglycemic effects [8,9,10,11,12].

To utilize the health benefits of Cordyceps militaris, we have prepared a spirit by soaking Cordyceps miliaris fruit into sweet potato shochu. In previous studies, its shochu was concentrated and passed through a synthetic adsorbent resin column. The bioactive fraction, eluted with 40% ethanol, was further separated by HPLC to fractionate the cordycepin [13,14]. However, the other active ingredient in the spirits remains unidentified, although the typic cordycepin is confirmed. It is essential to identify remaining compounds to understand the overall effect of Cordyceps militaris-infused spirits in sweet potato shochu.

Cordycepin, a major component of Cordyceps militaris, is an adenosine derivative. Adenosine has been reported to play an important role in tumor growth, differentiation, and apoptosis [15]. Extracellular adenosine regulates cellular activity, primarily through four G protein-coupled adenosine receptors. In particular, the A3 adenosine receptor (A3AR) has been found to be overexpressed in cancer cells. Thus, A3 receptors have been examined in several studies as a molecular target for cancer therapy [16,17]. Moreover, cordycepin was reported as an agonist of A3ARs and as exerting antitumor effects [18,19,20,21]. However, the ligand–receptor interactions underlying the mechanism of A3AR activation by adenosine derivatives remain to be elucidated.

Therefore, the present study aimed to characterize the chemical structure of the compounds that are present in Cordyceps militaris-infused spirits in sweet potato shochu and to verify their bioactivities. Firstly, the 40% ethanol-eluted spirit fractions, which were demonstrated to have in vitro anticancer activity in our previous study, were separated by HPLC. The in vitro anticancer activities of these fractions were then investigated in HL-60 and HCT-116 cell lines. Subsequently, the structures of these compounds that were present in the HPLC fractions were investigated by comprehensive chemical analyses including NMR, IR, and ESI-MS spectral analysis. Finally, the interactions of cordycepin and HEA, obtained from HPLC, with A3ARs were confirmed by using an A3AR antagonist and in silica molecular docking simulation. Our data will provide solid data on the chemical compounds in Cordyceps militaris-infused spirits in sweet potato shochu and their bioactivities. In our previous study, we demonstrated that soaking Cordyceps militaris in shochu produces a beverage containing the anticancer compound cordycepin. While the effects of soaking time and shelf life on the bioactivity of other liqueurs and wines are well known, their impact on Cordyceps militaris that is soaked in shochu remains unclear. Additionally, since Cordyceps militaris contains bioactive compounds besides cordycepin, it is crucial to investigate whether these compounds are also extracted during shochu soaking. In this study, we aim to verify the in vitro anticancer activity and bioactive compounds in Cordyceps militaris-infused sweet potato shochu spirits.

## 2. Results

### 2.1. Influence of Infusion Times and Storage Period on HCT-116 Cell Proliferation

**Figure 1:**
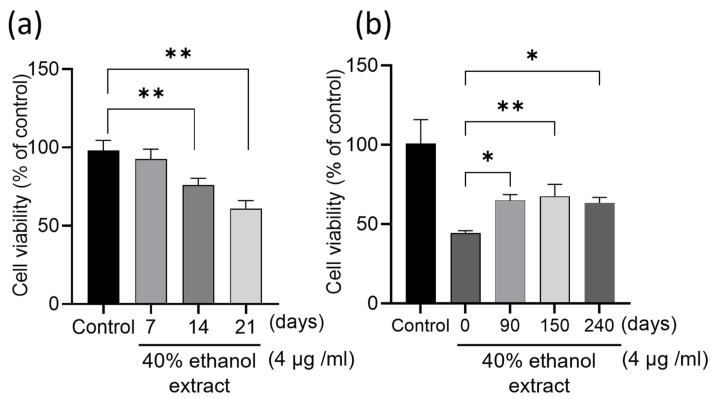
Inhibitory effect of infusion time (**a**) and storage period (**b**) of Cordyceps militaris-infused spirits on the proliferation of HCT-116 cells. The cells (2.2 × 10^4^ cells/cm^2^) were seeded in 96-well plates for 24 h and then treated for 48 h with indicated dose. Each value represents the mean ± S.D. of triplicate cultures. Columns with asterisk letters denote significant differences (* *p* < 0.05, ** *p* < 0.01).

To clarify whether the infusion times of Cordyceps militaris in sweet potato shochu affected its antitumor effect, Cordyceps militaris was infused in sweet potato shochu for 7, 14, and 21 days, respectively. The 21-day-infused spirits were further stored for 90, 150, and 240 days. All these spirits were concentrated to an extract and then eluted using 40% ethanol, which was proven as an effective solvent to obtain in vitro antitumor effect fractions in our previous study [13,14]. As shown in Figure 1a, a time-dependent inhibition in the proliferation of HCT-116 cells was observed in relation to the infusion times of Cordyceps militaris in sweet potato shochu from 7 to 21 days. For example, 75.9% cell viability was observed from treatment with 14 days of infusion of the extract, and 61% was observed from treatment with 21 days of infusion of the extract. Moreover, the storage period of the spirits also affected the inhibitory effect on the proliferation of HCT-116 cells. The fresh spirits (0-day storage) showed higher inhibitory effects than others that were stored for longer periods and maintained stable inhibitory effects from 90 to 240 days of storage at least (Figure 1b). These data suggest that the bioactive compounds in the fresh spirits may undergo structural changes during storage, although this was not clarified in this study.

### 2.2. Antitumor Effects of Fractions B to D on HCT-116 Cells

**Figure 2:**
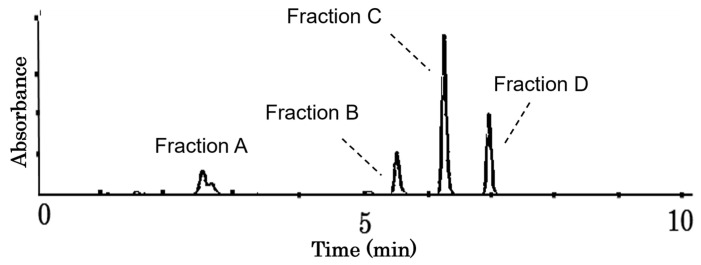
Chemical profile of fractions separated by HPLC-UV/Vis.

To clarify the compounds in the spirit extracts, the 40% ethanol extraction fraction underwent a single purification process, followed by separation into four sub-fractions using HPLC (C-18). As shown in Figure 2, four fractions (A–D) were separated, and their extraction ratios from 7.0 g of fruiting bodies were 0.016%, 0.0924%, 0.117%, and 0.0892%, respectively.

**Figure 3:**
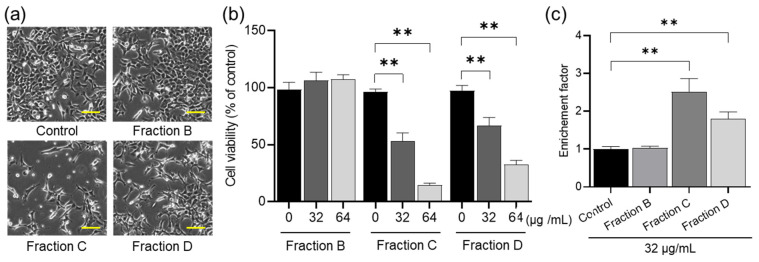
Morphological changes (**a**), proliferation inhibition effects (**b**), and DNA fragmentation induction (**c**) of HCT-116 cells by treatment with Fractions B to D. The cells (2.2 × 10^4^ cells/cm^2^) were seeded in 96-well plates for 24 h and then treated for 48 h with the indicated dose. The microscope magnification was 10× and the scale bar indicates 10.0 μm. The cell viability was measured by MTT assay. For the DNA fragmentation assay, the cells (3.7 × 10^3^ cells/cm^2^) were seeded into 12-well plates and then treated for 48 h with 32 μg/mL of the various fractions. The DNA fragmentation was detected using a Cell Death Detection ELISA^PLUS^ kit. Each value represents the mean ± S.D. of triplicate cultures. Columns with asterisk letters denote significant differences (** *p* < 0.01).

Due to the limited yield of Fraction A for the bioassay, we could only purify Fractions B–D for in vitro antitumor activity assays. During the preliminary experiment, we found that Fraction B had weak in vitro antitumor activity, while Fractions C and D had higher activity. To obtain an available dose for the cell assay, we chose a higher concentration of Fraction B. As shown in Figure 3, Fractions C and D significantly inhibited the proliferation of HCT-116 cells in the concentration range of 32–64 μg/mL in a dose-dependent manner (Figure 3a,b), but Fraction B did not. To further confirm whether the proliferation inhibition of HCT-116 cells is driven from apoptosis induction, the DNA fragmentation was examined using a Cell Death Detection ELISAPLUS kit. The DNA fragmentation caused by Fractions B–D showed the same results as their inhibitory effects on cell proliferation (Figure 3c).

### 2.3. Structural Identification of Compounds in Fractions B, C, and D

#### 2.3.1. Identification of Compounds in Fractions B, C, and D using ^1^H-NMR

It is known that adenosine, deoxyadenosine (cordycepin), and N^6^-(2-hydroxyethyl)-adenosine (HEA) are present in Cordyceps militaris [22]. To clarify whether these compounds were also present in the spirits, the structure of the compounds in Fractions B, C, and D were identified by ^1^H-NMR analysis. The ^1^H-NMR spectra of each compound are presented below.

**Figure 4:**
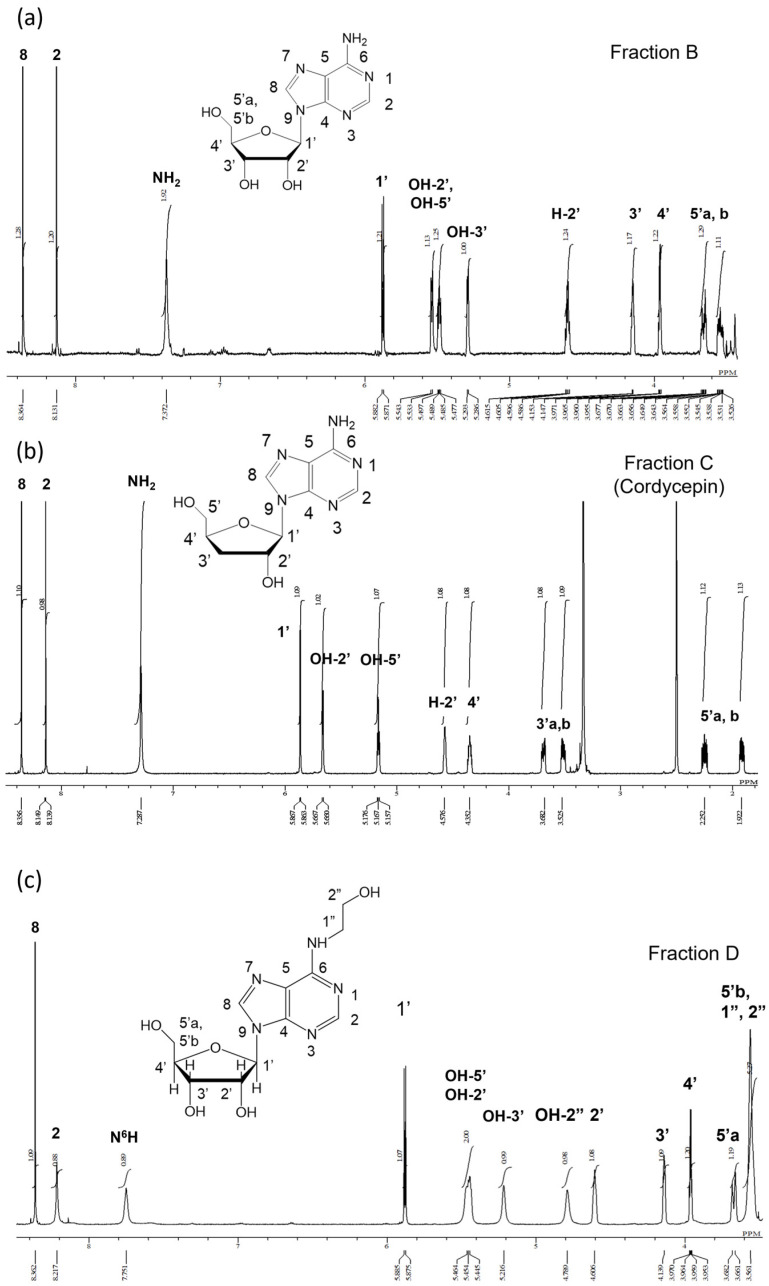
^1^H-NMR spectra of Fraction B (**a**), Fraction C (**b**), and Fraction D (**c**). Assignment based on ^1^H-NMR spectra of standards.

Fraction B in Figure 4a: ^1^H-NMR (600 MHz, DMSO-d6): δ8.36 (1H, s, H-8), 8.13 (1H, s, H-2), 7.37 (2H, brs, NH_2_), 5.88 (1H, d, *J* = 6.6 Hz, H-1′), 5.54 (1H, d, *J* = 4.4 Hz, OH-2′), 5.48 (1H, q, *J* = 4.8, 7.2 Hz, OH-5′), 5.29 (1H,d, *J* = 4.7 Hz, OH-3′), 4.60 (1H, m, H-2′), 4.15 (1H, d, *J* = 3.0 Hz, H-3′), 3.96 (1H, d, *J* = 3.0 Hz, H-4′), 3.66/3.53 (1H, m, H-5′). Compared with the ^1^H-NMR spectrum of adenosine (Figure S1a) and published data in Ref. [23], the ^1^H-NMR spectrum of Fraction B was in agreement with adenosine.

Fraction C in Figure 4b: ^1^H-NMR (600 MHz, DMSO-d6): δ8.35 (1H, s, H-8), 8.13 (1H, d, *J* = 6.0 Hz, H-2), 7.28 (2H, brs, NH_2_), 5.86 (1H, t, *J* = 6.6 Hz, H-1′), 5.66 (1H, d, *J* = 4.4 Hz, OH-2′), 5.19 (1H, t, *J* = 5.4, 6.0 Hz, OH-5′), 4.56 (1H, s, H-2′), 4.33 (1H, s, H-4′), 3.67/3.53 (1H, m, H-5′), 2.23/1.91 (1H, s, H-3′). Compared with the ^1^H-NMR spectrum of cordycepin and published data in Ref. [24], the ^1^H-NMR spectrum of Fraction C was in agreement with cordycepin.

Fraction D in Figure 4c: ^1^H-NMR (600 MHz, DMSO-d6): δ = 8.36 (1H, s, H-8), 8.21 (1H, s, H-2), 7.75 (1H, brs, N^6^H), 5.88 (1H, d, *J* = 6.0 Hz, H-1′), 5.45 (2H, t, OH-2′, OH-5′), 5.21 (1H, s, OH-3′), 4.78 (1H, bs, OH-2″), 4.61 (1H, brs, H-2′), 4.13 (1H, s, H-3′), 3.96 (1H, dd, *J* = 6.6, 3.6 Hz, H-4′), 3.67 (1H, d, *J* = 12.6 Hz, H-5′a), 3.56 (5H, s, H-5′b, H-1″, H-2″). To confirm whether the signal at 4.78 ppm originated from OH-2″, a drop of D_2_O was added to a sample dissolved in a DMSO-d6 solvent and stirred. The proton exchange with D2O resulted in broadening of the already assigned peaks at 7.75 ppm (N^6^H), 5.45 ppm (OH-2′, OH-5′), and 5.21 ppm (OH-3′). Similarly, the signal at 4.78 ppm was associated with OH-2″, as evidenced by its significant reduction (Figure S2).

The structures of these fractions were determined by comparing the ^1^H-NMR spectra of each compound with the ^1^H-NMR spectra of standard adenosine, cordycepin, and HEA (Figure S1a–c). The ^1^H-NMR spectra of Fractions B and C completely matched the ^1^H-NMR spectra of adenosine and cordycepin, respectively. Therefore, Fractions B and C were identified as adenosine and cordycepin, respectively. Although the ^1^H-NMR spectrum of Fraction D (Figure 4c) was consistent with the spectrum of the standard compound HEA (Figure S1c), both showed discrepancies with published data [22,25] in their chemical shifts and integration ratios. Specifically, the OH-2′ and OH-5′ peaks and the H-5′b, H-1″, and H-2″ peaks were observed as a combined single peak. Moreover, a single peak combining the H-5′b, H-1″, and H-2″ peaks had an estimated integration ratio of 3, while the observed value was 6. Thus, the ^1^H-NMR spectra of Fraction D were not sufficient to prove that Fraction D was HEA.

#### 2.3.2. Structural Analysis of Fraction D Using ^13^C-NMR, FT-IR, and ESI-MS

**Figure 5:**
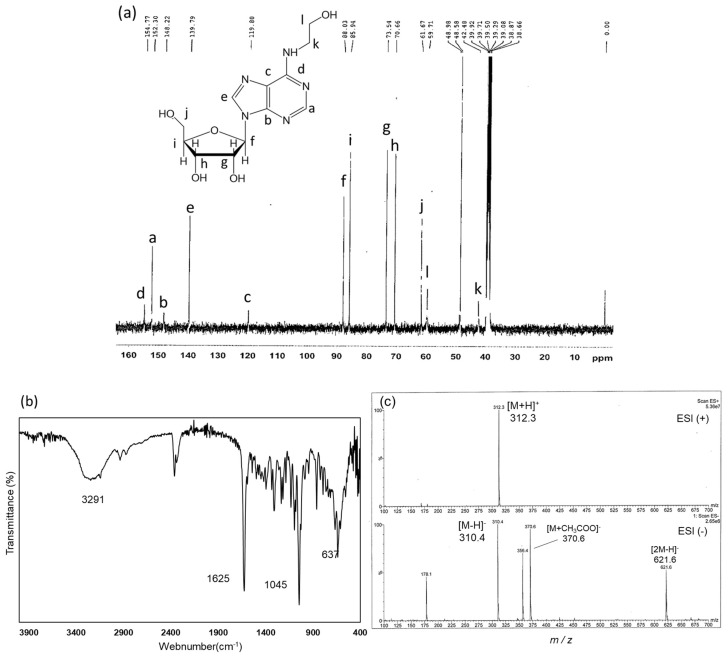
Comprehensive structural analyses of Fraction D. (**a**) ^13^C−NMR spectrum, (**b**) FT−IR spectra, and (**c**) ESI−MS spectrum (positive ion mode: upper panel and negative ion mode: bottom panel) of Fraction D.

To further identify Fraction D, ^13^C-NMR, FT-IR spectral, and electrospray ionization–mass spectrometry (ESI-MS) analyses were performed. ^13^C-NMR (125 MHz, DMSO-d6): δ154.7 (C-6), 152.2 (C-2), 148.1 (C-4), 139.7 (C-8), 119.6 (C-5), 88.0 (C-1′), 85.8 (C-4′), 73.4 (C-2′), 70.5 (C-3′), 61.6 (C-5′), 59.7 (C-2″), 42.4 (C-1″). Compared with published standard data, the ^13^C-NMR chemical shifts of Fraction D were consistent with HEA (Figure 5b). The FT-IR spectra (Figure 5c) show a broad single absorption for O-H at 3291 cm^−1^, with stretching vibrations. The absorption bands at 2938 cm^−1^ were attributed to C–H stretching vibrations. A specific peak for the C=N group appeared with a strong band in the expected region at 1625 cm^−1^, while the C-O-C group also appeared at 1045 cm^−1^. The IR spectrum showed data that were in perfect agreement with the standard sample. In addition, as shown in Figure 5d, the ESI-MS analysis showed that the molecular weight of Fraction D was consistent with the molecular weight of HEA: ESI-MS (positive ion mode) *m*/*z:* 312.3 [M+H]^+^, and ESI-MS (negative ion mode) *m*/*z*: 310.4 [M-H]^−^ (calcd for C_12_H_17_N_5_O_5_, 311.29). The overall interpretation of these analyses confirmed that Fraction D was HEA.

### 2.4. Interaction between Human Adenosine A3 Receptors (A3ARs) and Adenosine, Cordycepin, and HEA

#### 2.4.1. Cordycepin and HEA Inhibit HCT-116 Cell Proliferation as A3AR Agonists

**Figure 6:**
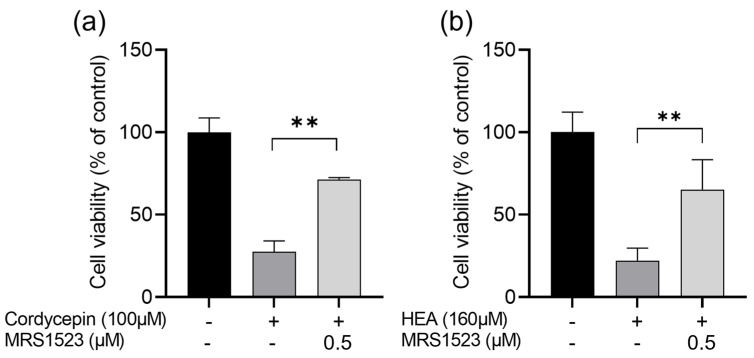
Effect of adenosine receptor antagonist on cordycepin (**a**) and HEA (**b**)−induced cell proliferation inhibition in HCT−116 cells. MR1523 was used as A3AR antagonist. Each value represents mean ± S.D. of triplicate cultures. Columns with asterisk letters denote significant differences (** *p* < 0.01).

Both cordycepin and HEA are adenosine derivatives. It has been reported that adenosine can activate A3ARs to inhibit cell proliferation [26,27]. To clarify whether cordycepin and HEA also serve as A3AR ligands to inhibit cell proliferation in HCT-116 cells, we examined the competition of cordycepin and HEA with an A3AR-specific antagonist, MRS1523. As shown in Figure 6, treatment of HCT-116 cells with cordycepin (100 μM) and HEA (160 μM) for 48 h reduced the cell viability to 27.6% and 22.2%, respectively, compared to non-treated controls. When pretreated with MRS1523 (0.5 μM), an A3AR-specific antagonist, the cell viability was significantly recovered, up to 71.3% and 65.2%, respectively.

#### 2.4.2. Prediction of Binding Site and Energy on A3AR via MOE-ASEDock

**Figure 7:**
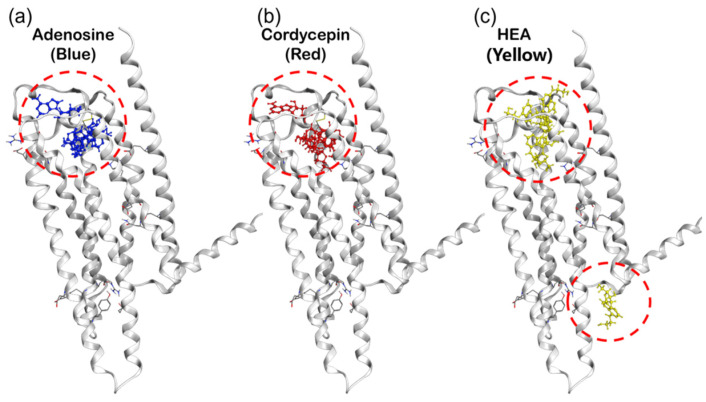
Prediction of the binding region between human A3AR and adenosine (**a**), cordycepin (**b**), or HEA (**c**). (**a**–**c**): Docking simulations using the whole A3 molecule as a binding target. The top five poses predicted to bind using MOE−Dock are overlaid on the human A3AR structure model (gray color). Blue indicates adenosine, red indicates cordycepin, and yellow indicates HEA. Their binding sites for the five poses are circled in red.

To examine whether cordycepin and HEA are possible agonists of the A3AR, in silico binding simulations of adenosine, cordycepin, and HEA with A3ARs were performed by MOE-ASEDock soft. The calculations revealed that both adenosine and cordycepin bind to the predicted space pocket in all top 5 poses of the 300 binding-generating poses (Figure 7a,b). In contrast, HEA exhibited a different binding pattern, of which two of the top five poses were predicted to bind to the same region as adenosine and cordycepin, while another two poses were anticipated to bind to a larger spatial area on the extracellular domain side (Figure 7a–c).

**Figure 8:**
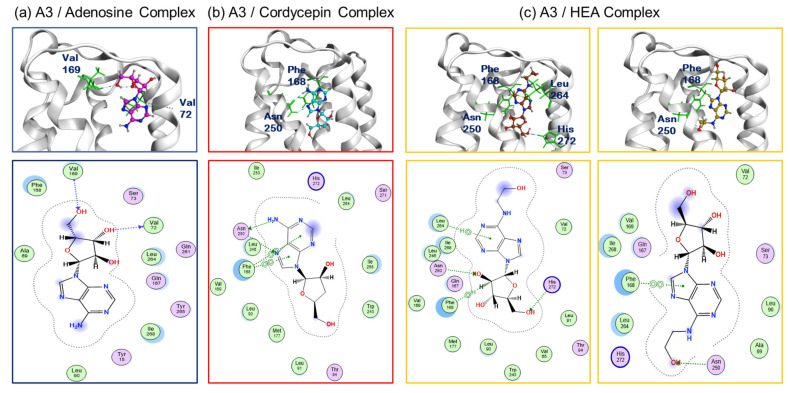
Prediction of binding between human A3AR and adenosine (**a**), cordycepin (**b**), or HEA (**c**). Top: Three-dimensional protein poses with their binding sites displayed. Bottom: Two-dimensional view displaying the docking poses of the major compound residues interacting with protein interactions in relation to the top figure. The indicated docking simulation diagram is a typical example from among five simulation poses.

The quantum chemical analysis of the A3AR binding site reveals that adenosine and cordycepin share the same binding region and demonstrate similar binding frequencies. However, their respective abilities to induce apoptosis differ significantly. Therefore, our focus shifted to examining the binding pattern within the endogenous ligand-binding pocket (Figure 8a–c) rather than the entire A3 molecule’s binding. When interacting with different ligands in the top five binding positions, distinct binding patterns were observed for adenosine, cordycepin, and HEA.

For adenosine, all top five poses featured an imidazole ring, oriented within the binding pocket. These positions exhibited strong interactions with the amino acid side chain of the A3 receptor at the hydroxy group of the ribose. In contrast, for cordycepin, four out of the five poses consistently showed the imidazole ring being oriented towards the exposed side of the binding pocket, unlike adenosine. These poses demonstrated robust interactions between the cordycepin imidazole ring and the aromatic amino acid Phe168 in the A3AR. Notably, the presence of a hydroxy group at position 3 of the ribose significantly influenced the binding pattern.

In the case of HEA, the analysis of binding patterns within the pocket revealed a mixture of orientations. Three poses resembled adenosine, with the imidazole ring being oriented internally, while two poses resembled cordycepin, with the imidazole ring being oriented externally. This diversity not only highlights variations in the binding region (see Figure 7c) but also in the binding pattern itself. These results suggest that the relatively lower apoptosis-inducing capacity of HEA, despite its higher affinity for A3ARs, may be attributed to the diversity of binding patterns and the dispersion of the binding region.

## 3. Discussion

### 3.1. Effect of Soaking Time and Storage Period on Bioactivity of Shochu Spirits

The separation and purification of cordycepin from Cordyceps militaris have been extensively documented through column chromatography. Quan et al. provided a simple protocol approach for cordycepin isolation and purification, obtaining it from fruiting bodies of Cordyceps militaris with a yield of 0.04% through dual-normal-phase column chromatography [28]. Zhang et al. obtained cordycepin at a yield of 0.094%, along with adenosine and HEA at yields of 0.14% and 0.10%, respectively, through the use of macroporous resin and high-speed counter-current chromatography with the fruiting bodies [22]. We achieved a cordycepin extraction rate of 0.117% through a two-step purification process involving immersion in shochu (Japanese distilled spirit), a Diaion HP-20 column, and C-18 HPLC. Furthermore, adenosine and HEA were obtained in yields of 0.0924% and 0.0892%, respectively. Compared to the results reported by Zhang et al., the yield of cordycepin in this study was higher, although the yields of adenosine and HEA were inferior. These data indicate that Cordyceps militaris-infused spirits in soaked sweet potato shochu can efficiently obtain cordycepin with a higher bioactivity.

The infusion time and storage period affected the in vitro anticancer activity. These data suggested that the bioactive compounds, such as adenosine, cordycepin, and HEA, most likely need time to be eluted out from Cordyceps militaris to the spirits when using a low-alcohol-concentration sweet potato shochu. Regarding the storage period, the fresh spirit extract showed higher in vitro anticancer activity, which was reduced slightly by 90 days of storage. It then remained stable for at least 240 days. These data suggest that the bioactive compounds in the fresh spirits may undergo structural changes during storage, although this was not clarified in this study. It should be noted that certain compounds in fruit liquors and wines can change during storage. For example, phenolic compounds such as catechins and anthocyanins have been reported to undergo chemical changes during storage. These changes were suggested to affect storage stability and antioxidant capacity [29,30,31]. For example, the antioxidant activity of cherry liqueur was reported to be reduced to 37.8% after six months of storage at 30 °C and correlated strongly with the content of flavonols, anthocyanins, flavanols, and phenolic acids [29]. The reduced inhibitory effect of Cordyceps militaris-infused spirits on cell proliferation during the 90-day storage period is possibly related to the reduction in phenolic compounds. Future studies should quantitatively analyze structural changes and their content levels in bioactive compounds, particularly in cordycepin, to correlate them with the changes in in vitro anticancer activity during storage.

### 3.2. Interaction of Adenosine, Cordycepin, and HEA with A3ARs

#### 3.2.1. The Relationship between Cordycepin or HEA and A3ARs in Inhibiting Cell Proliferation

Previous research has identified cordycepin as an A3AR ligand, and its activation has been linked to the inhibition of proliferation and induction of apoptosis in some cancer cells [18,20,21]. HEA is also an adenosine derivative that has been reported to have antitumor effects [32]. In particular, the present results suggested that HEA has a more potent antitumor effect than adenosine. However, whether HEA is an agonist of A3AR has not been established. Thus, we examined the effect of HEA and cordycepin on the proliferation of HCT-116 cells by an A3AR agonist test. Our data revealed that the inhibitory effects of cordycepin and HEA on HCT-116 cell proliferation were reversed by MRS1523, an A3 adenosine receptor antagonist. This suggests that the inhibitory effects of cordycepin and HEA might be mediated through A3AR. The modification of the adenosine moiety at the N6 position has been reported to influence the selectivity of A1AR and A3AR [33]. CF101, an adenosine-derived A3AR agonist, synthesized with a similar N6 position modification that was found to inhibit cell proliferation; furthermore, this effect of CF101 was reversed by the selective A3AR antagonist MRS1523 in HCT-116 cells [34]. In this study, HEA was also modified at the N6 position of the adenosine moiety, and its antiproliferation was reversed by MRS1523. These data suggested that HEA inhibits cell proliferation as an A3AR agonist. This observation is also fully consistent with the findings of Yoshikawa et al., who demonstrated the involvement of adenosine A3 receptors in the action of cordycepin using specific antagonists, MRS1523 and MRS1220 [19] and MRS1191 [20]. Additionally, Cao et al. showed that a hot water extract of Cordyceps militaris containing cordycepin induced caspase-3 activity and apoptosis to decrease the survival of human bladder cancer cells (T24 cells) by activating A3ARs and subsequently deactivating the Akt pathway [21]. Therefore, our data combined with previous reports indicate that both cordycepin and HEA act as agonists of A3ARs to inhibit HCT-116 cell proliferation.

#### 3.2.2. In Silico Analysis of the Interaction of A3AR with Cordycepin and HEA

Yoshikawa et al. reported the binding of cordycepin to adenosine A3 receptors in B16-BL6 cells through a radioligand binding assay using [125I]-AB-MECA, a selective adenosine A3 receptor agonist [19]. However, due to the lack of a crystal structure of A3ARs, there has been no report on the simulation of the binding site between A3ARs and cordycepin. Therefore, we first constructed a predicted structure of A3ARs. The predicted structure of the human A3AR that was employed for the docking analysis closely resembles the crystal structures of human A2AR (PDB number: 3EML) [35] and human A1AR (PDB number: 5UEN) [36], with significantly higher homology in the transmembrane region. The only structural disparities between the predicted A3 structure and A1 and A2 were found in the loop-and-turn structures within the extracellular domain region, which is a flexible area. The binding regions with the endogenous ligand adenosine have been elucidated in A1 and A2. Docking simulations with adenosine targeting these binding sites yielded E-scores (kcal/mol) of −10.1010 for A1AR and −9.6427 for A2AR. Comparatively, aligning the structures of A1 and A2 with the predicted A3 structure reveals a spatial pocket in the same region as the ligand-binding regions of A1 and A2. Consequently, we targeted this space pocket and conducted adenosine docking with A3. The E-scores (kcal/mol) for the top five binding poses ranged from −10.0376 to −10.9344, which were similar to those obtained for A1 and A2. Based on these calculations, the selected A3 structure was deemed appropriate for the analysis, and we subsequently performed docking simulations of A3 with other ligands (cordycepin and HEA).

The binding energy (E-score), which serves as a benchmark for evaluating docking simulations of various ligands with A3, indicated that HEA tended to have higher E-scores compared to adenosine, while cordycepin exhibited lower E-scores than adenosine. However, these differences were statistically insignificant (Figure S3). This variance in E-scores was presumed to be a result of structural influences originating from the ligand’s functional group. In adenosine derivatives, small N6-alkyl substitutions on the imidazole ring have been reported to be associated with selectivity for human A3ARs over rat A3ARs [37], as well as an increased affinity toward A3ARs [38]. HEA is substituted with hydroxy adenosine with a hydroxy group at the end of this small alkyl chain, which may explain its higher affinity for A3ARs than its parent compound, adenosine.

### 3.3. Differences in Proliferation Inhibition by Adenosine, Cordycepin, and HEA in HL-60 and HCT-116 Cells

In our previous report, we reported that cordycepin induced apoptotic cell death via the caspase pathway in HL-60 human leukemia cancer cells and HCT-116 human colon cancer cells. In this study, we evaluated the ability of adenosine and HEA to induce apoptosis in both cells. The results showed that HEA induced significant apoptosis in both cell types, although the induction ability was weaker than that of cordycepin. Adenosine did not induce apoptotic cell death in adherent HCT-116 cells, but it did induce apoptosis in floating HL-60 cells, with a more pronounced inhibitory effect on cell growth than the control (Figure S4). HL-60 cells were found to exhibit cell growth inhibition and apoptosis induction in response to adenosine cordycepin and HEA at lower concentrations compared to HCT-116 cells. This finding is consistent with previous results involving compounds such as dihydroartemisinin [39], bilberry anthocyanin [40], and 9,10-phenanthrenequinone [41], where HL-60 cells were demonstrated to be more sensitive with respect to cell growth inhibition and apoptosis induction at lower concentrations compared to HCT-116 cells. Adherent cells can receive signals that are necessary for survival and proliferation through physical contact with other cells and substrates [42,43]. Floating cells have reduced contact with other cells and the extracellular matrix compared to adherent cells, leading to decreased intercellular signaling. This makes them less resistant to apoptosis-inducing factors and more sensitive to such stimuli. An elevated expression of the A3 adenosine receptor has been demonstrated in cancer cells from colon cancer patients and is reflected in peripheral blood cells [44]. Its A3 adenosine receptor stimulation has been shown to inhibit tumor growth in leukemia, colon cancer, lymphoma, and pancreatic cancer [16]. These reports, together with the results shown in Figure 7, support the notion of an A3 adenosine receptor-mediated inhibition of cell growth by adenosine and cordycepin and HEA.

Shochu is a popular alcoholic beverage among many Japanese people. Cordyceps militaris is a representative species that has traditionally been used in Chinese medicine and as a medicinal food. In this study, the in vitro anticancer activity and bioactive compounds of Cordyceps militaris-infused sweet potato shochu spirits were first investigated, and the data revealed that the bioactive compounds, including cordycepin, HEA, and adenosine, were present in the spirits and contributed to in vitro anticancer activity. Although the result was limited at the cultured cell-level and future animal or human studies are needed to confirm these results, this study indicates that shochu spirits that are infused with Cordyceps militaris may be a helpful step to developing healthy shochu sprits compared to regular alcoholic liquids.

## 4. Materials and Methods

### 4.1. Reagents

3-(4,5-dimethylthiazol-2-yi)-2,5-diphenyltetrazolium bromide (MTT) from Sigma-Aldrich (Burlington, MA, USA) was used for measuring cell proliferation potential. Cordyceps militaris fruiting bodies were produced in South Korea (Mushtech Co., Ltd., Gangwon, Republic of Korea), dried at 45 °C, 55 °C, and 65 °C in stages and frozen at −25 °C. Sweet potato shochu was produced by Kirishima Shuzo Co., Ltd. (Miyazaki, Japan); Adenosine (>98.0%) was purchased from Tokyo Chemical Industries (TCI, Tokyo, Japan). 3′-Deoxyadenosine (Cordycepin (>98.0%)) was produced by Fujifilm Wako Chemicals (Osaka, Japan). N^6^-(2-hydroxyethyl)-adenosine (>99.0%) was purchased from GlpBio Technology (Montclair, CA, USA). 3-propyl-6-ethyl-5-[(ethylthio)carbonyl]-2 phenyl-4-propyl-3-pyridine carboxylate (MRS1523, adenosine A3 receptor antagonist) was purchased from Sigma Chemical (St. Louis, MO, USA). Rabbit Polyclonal Adenosine A3 Receptor antibody (GTX131656) was purchased from Gene Tex (Los Angeles, CA, USA).

### 4.2. Preparation of Cordyceps Militaris-Infused Spirits in Sweet Potato Shochu and Fractionation of Their Extracts

Seven grams of Cordyceps militaris fruiting bodies was added to 1000 mL of sweet potato shochu with an alcohol concentration of 36%. After maceration and extraction at room temperature for 3 weeks, the mixture was filtered through a 0.45 µm filter, and the supernatant was collected. The collected supernatant was diluted with water to 20% ethanol solution and passed through a Diaion HP20 synthetic absorption resin column (Mitsubishi Chemical, Tokyo, Japan). Fractions eluted with 40% ethanol were concentrated to 50 mL at 40 °C using a rotary evaporator and then freeze-dried (Freeze Dryer DC41, Yamato Scientific, Tokyo, Japan). The fraction was gel-filtered through Sephadex G-25 (GE Healthcare Japan, Tokyo) and was further separated by HPLC (C-18) into Fractions A, B, C, and D. HPLC was performed using a Shimadzu LC-20A system under the following conditions: Pump: LC-20ADVP; oven column: CTO-20AC; UV/Vis detector: SPD-20A; autosampler: SIL-20AHT; column: Cadenza CD-C18 (3 µm, 75 × 4.6 mm I.D., Imtakt Co., Kyoto, Japan); column temperature: 40 °C; mobile phase: (A) water, (B) acetonitrile; flow rate: 1.0 mL/min, 2 to 19% B (0.00 to 8.00 min), 19 to 100% B (8.01 to 16.00 B (8.01 to 16.00 min), 100% B (16.01 to 20.00 min), and 2% B (20.01 to 26.00 min). The detection wavelength was set to 260 nm. These fractions were freeze-dried and used as samples in this study.

### 4.3. Cell Culture

Human colorectal cancer cell lines HCT-116 were obtained from the American Type Culture Collection (Manassas, VA, USA). The cells were cultured at 37 °C in a 5% CO_2_ atmosphere in Dulbecco’s Modified Eagle Medium containing 10% fetal bovine serum and 1% of penicillin–streptomycin glutamine for 24 h, and then treated by each fraction using indicated times and doses. Human promyelocytic leukemia cells (HL-60) were obtained from RIKEN CELL BANK (Saitama, Japan). The cells were cultured in RPMI 1640 medium (NISSUI PHARMACEUTICAL CO., Tokyo, Japan) containing 10% FBS (Equitech-Bio Inc., Kerrville, TX, USA) and 1% PS-L-glutamine mixture (PSG) (GIBCO Inc., Burlington, MA, USA) at 37 °C under 5% CO₂ conditions for 24 h, and then treated by each fraction using indicated times and doses.

### 4.4. Cell Viability Assay

Cell viability was determined by an MTT assay as described previously. Briefly, HCT-116 cells or HL-60 cells were plated in each well of 12- or 96-well plates, respectively. The cells were treated with Fractions B, C, and D for 48 h. MTT solution was then added to each well and incubated for another 4 h. The resulting MTT-formazan product was dissolved in 100 μL of 0.04 N HCl-2-propanol and determined by measuring the absorbance at 595 nm with Multiskan TM FC (Thermo ScientificTM, Waltham, MA, USA). The cell viability was expressed as the optical density ratio of the treatment to control.

### 4.5. Identification of Chemical Compounds in Spirit Extract Fraction

Fraction B and Fraction D were analyzed by ^1^H-NMR, which was recorded on a spectrometer (JEOL-ECA600, 600 MHz, JEOL, Tokyo, Japan) using DMSO-d6 as solvents and tetramethylsilane (TMS) as internal standard. Chemical shifts and J values are given in Hz. The ^13^C-NMR spectra of Fraction D were also recorded using a Bruker UltraShield 400 MHz NMR spectrometer (Bruker Corporation, Billerica, MA, USA). All samples were dissolved in DMSO-d_6_ and measured (2 mg/0.7 mL for ^1^H-NMR, 20 mg/0.7 mL for ^13^C-NMR). Furthermore, the structure was comprehensively determined by FT-IR and electrospray ionization mass spectrometry (ESI-MS) measurements. ESI-MS spectra were acquired using an Esquire 3000 plus instrument (Agilent Technologies, Inc., Santa Clara, CA, USA). The ESI-MS conditions were as follows: positive or negative ion mode, injection flow rate of 2 μL/min, drying gas (nitrogen) flow rate of 4 L/min, nebulizer pressure of 10 psi (equivalent to 29.3 kgf/cm^2^), drying gas temperature of 300 °C, and a mass scan range of 50–1000 *m*/*z*. FT-IR/IRT-3000 ATR-30-Z (JASCO) was used for identification. Attenuated Total Reflection (ATR) was performed by placing a powder sample over the entire surface of the ATR crystals and then pressing the sample firmly against the prism while compressing it. The measuring range was 400–4000 cm^−1^.

### 4.6. ELISA Detection of DNA Fragmentation

Cell Death Detection ELISA^PLUS^ (Roche Diagnostics, Basel, Switzerland) was used to determine DNA fragmentation according to the manufacturer’s instructions. HCT-116 cells (3.7 × 10^3^ cells/cm^2^) or HL-60 cells (1.0 × 10^5^ cells/mL) were plated in 12-well plates and treated with each sample. After lysing the cells with lysis buffer and centrifuging at 200× *g* for 10 min, the supernatant was added to each well of the ELISA plate and incubated with immunoreagent buffer containing anti-histone biotin and anti-DNA POD for 2 h. Following washing with incubation buffer, 100 L of ABTS substrate buffer was added, and the absorbance was measured at 405 and 490 nm using Multiskan^TM^ FC (Thermo ScientificTM, Waltham, MA, USA). The optical density ratio of the treatment to the control was used to calculate the enrichment factor of the apoptosis induction.

### 4.7. Adenosine Receptor Antagonist Assay

The interaction between cordycepin or HEA and A3ARs was investigated by examining the cell viability with an A3AR-specific antagonist (MRS1523). HCT-116 (7.9 × 10^4^/well) cells were plated into each well of 12-well plates and pretreated with MRS1523 (0.5 μM) for 30 min. Cordycepin (100 μM) or HEA (160 μM) was then added to the wells. After 48 h of incubation at 37 °C, viable cells were counted on a TC20 automated cell counter (Bio-Rad Laboratories, Inc., Hercules, CA, USA).

### 4.8. In Silico Simulation of the Interaction between Adenosine, Cordycepin, and HEA and Human A3ARs

The predicted structure from the AlphaFold DB (ID: AF-P0DMS8) [1] was used in this study, since the crystal structure of human A3AR has not been determined. The obtained structure was optimized using Molecular Operating Environment (MOE version 2022, Chemical Computing Group) for docking simulations. The structure of ligands (Adenosine, Cordycepin, and HEA) were generated with chemdraw and optimized using MOE. Compound docking analysis was performed using the MOE-Dock program. The binding sites were targeted to the whole molecule, including known pockets, and the force field was performed with MMFF94. The binding methodology was adapted to fix the receptor, and the ligand could move upon binding. As for docking assessments, 300 candidate docking poses were generated and evaluated using the London dG score (E-score, kcal/mol), which estimates the binding free energy of the ligand from a given pose to filter them down to the top five poses.

### 4.9. Statistical Analysis

The results of each experiment are presented as mean ± standard deviation. Significant difference tests were performed with the statistical software “GraphPad Prism version 9.5.1, GraphPad Software, San Diego, CA, USA, www.graphpad.com (accessed on 1 February 2024)”. MTT (*n*= 8) and ELISA (*n*= 3) measurements were performed at least three times each, and one-way analysis of variance (ANOVA) followed by Tukey’s multiple comparison was performed for significance testing.

## 5. Conclusions

Adenosine, cordycepin, and HEA are presented in Cordyceps militaris-infused shochu spirits as bioactive compounds. These compounds revealed in vitro anticancer effects on HCT-116 and HL-60 cells following the order of cordycepin, HEA, and adenosine.

## Data Availability

Data are contained within the article and its Supplementary Information file.

## Supplementary Materials

The following supporting information can be downloaded at https://www.mdpi.com/article/10.3390/molecules29092119/s1, Supplementary Figure S1: ^1^H-NMR spectrum of (a) Adenosine and (b) HEA in DMSO-d_6_ solvent; Figure S2: ^1^H-NMR spectrum of HEA in DMSO-d_6_ solvent (top panel) and spectrum of DMSO-d_6_ solution sample with one drop of D_2_O (bottom panel); Figure S3: Top five poses predicted to bind using MOE-Dock for the human A3AR structural model and their respective binding energies (E-score); Figure S4: The effect of Cordyceps militaris-infused spirits on the proliferation inhibition and apoptotic induction of HL-60 cells.
