# Supplementary material for: Verification of In Vitro Anticancer Activity and Bioactive Compounds in Cordyceps Militaris-Infused Sweet Potato Shochu Spirits
Source: Molecules. 2024 May 3;29(9):2119. doi: 10.3390/molecules29092119 (PMC11085083; doi:10.3390/molecules29092119)
Supplement: Supplementary file 1 [file molecules-29-02119-s001.zip › molecules-2976139-supplementary.pdf]

Supplementary Figures

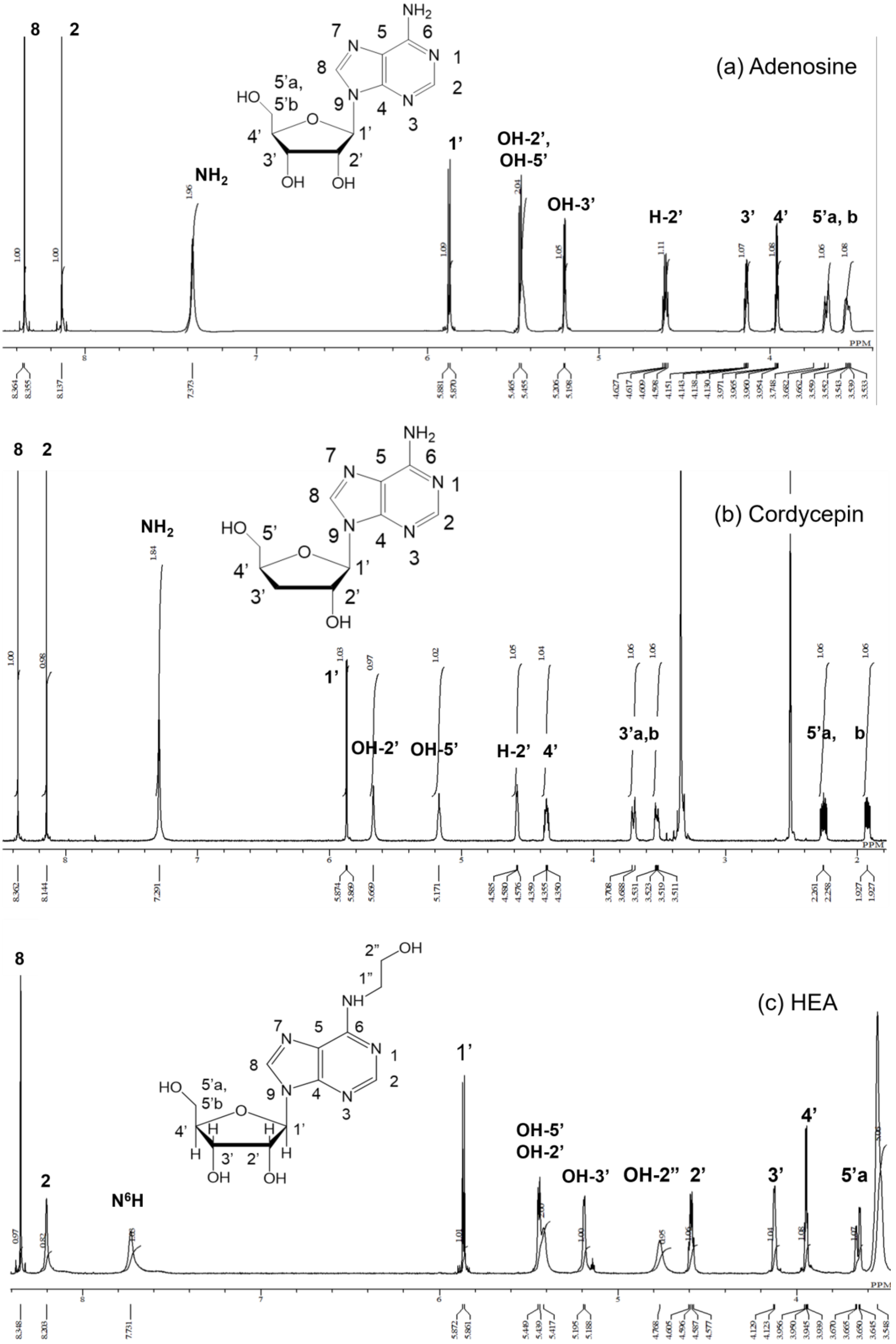

Figure S1. ^{1}H-NMR spectrum of (a) Adenosine, (b) Cordycepin and (c) HEA in DMSO-d_6 solvent

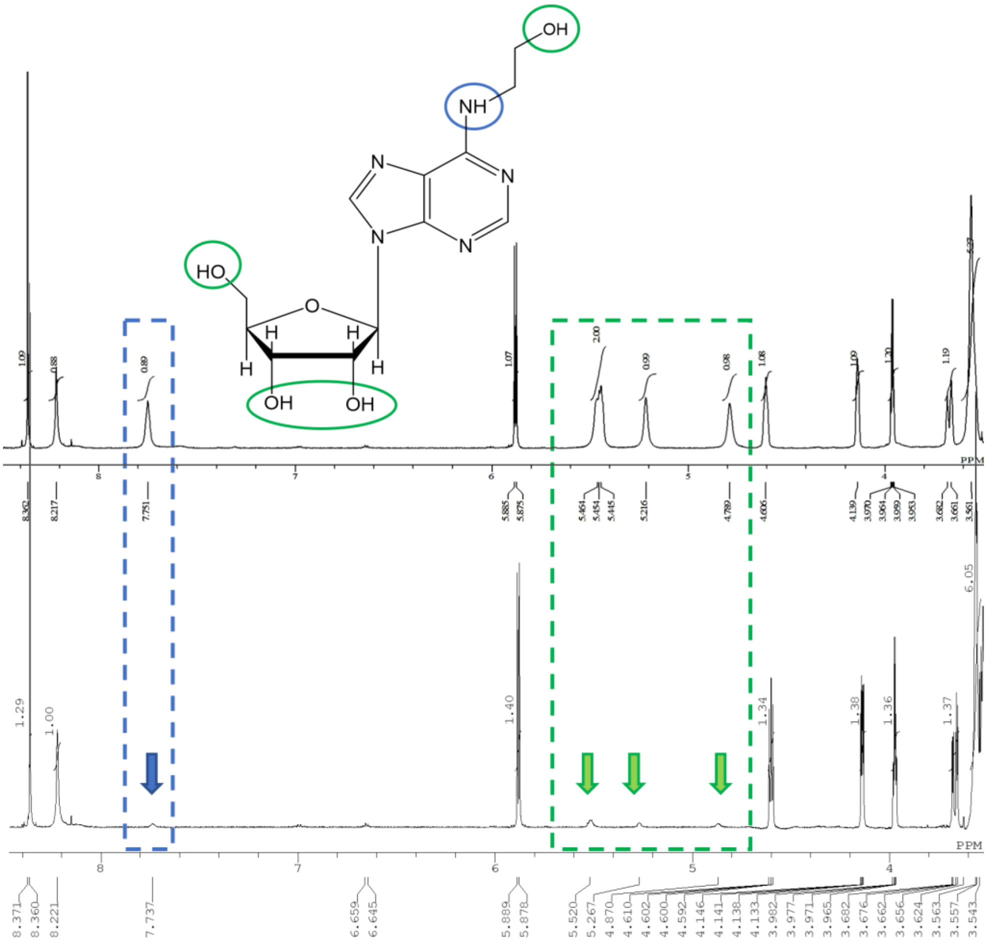

Figure S2. ^{1}H-NMR spectrum of HEA in DMSO-d_6 solvent (top panel) and spectrum of DMSO-d_6 solution sample with one drop of D_2O (bottom panel)

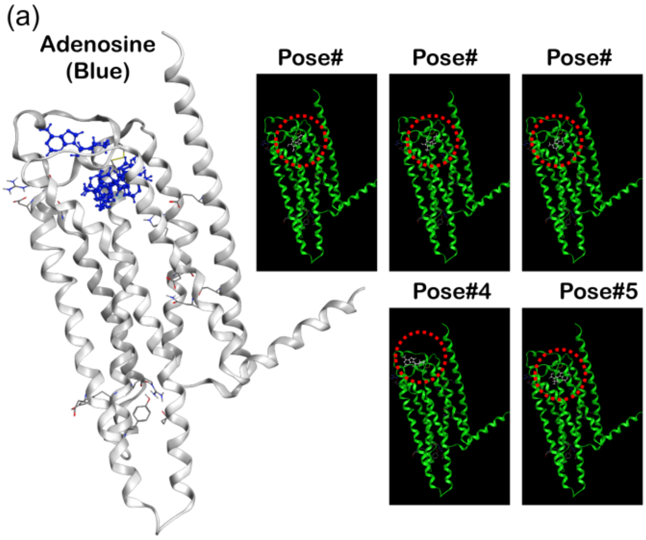

Pose	E-Score (kcal/mol)
#1	-7.2093
#2	-7.8372
#3	-8.8196
#4	-7.4854
#5	-8.0121

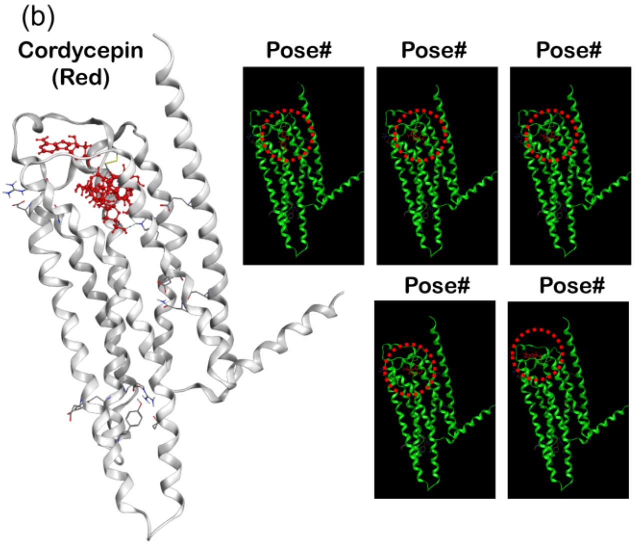

Pose	E-Score (kcal/mol)
#1	-7.2685
#2	-7.4683
#3	-7.1791
#4	-7.2984
#5	-7.6865

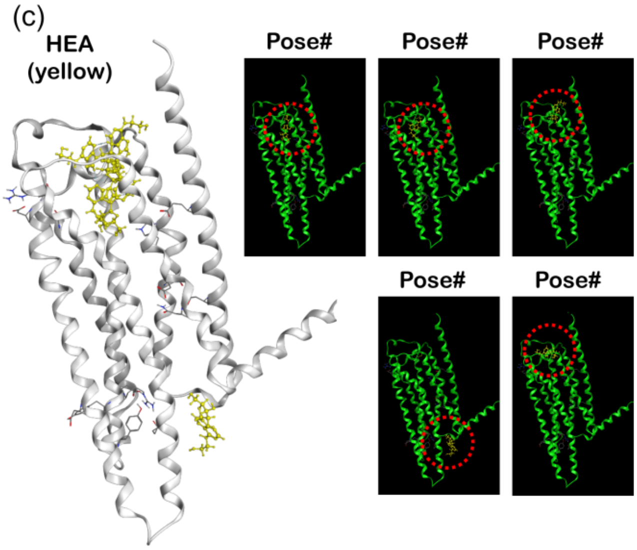

Pose	E-Score (kcal/mol)
#1	-8.6094
#2	-8.5336
#3	-8.7939
#4	-9.4357
#5	-8.6601

Figure S3. Top 5 poses predicted to bind using MOE-Dock for the human A3AR structural model and their respective binding energies (E-score). (a), (b), and (c) indicate adenosine, cordycepin, and HEA, respectively.

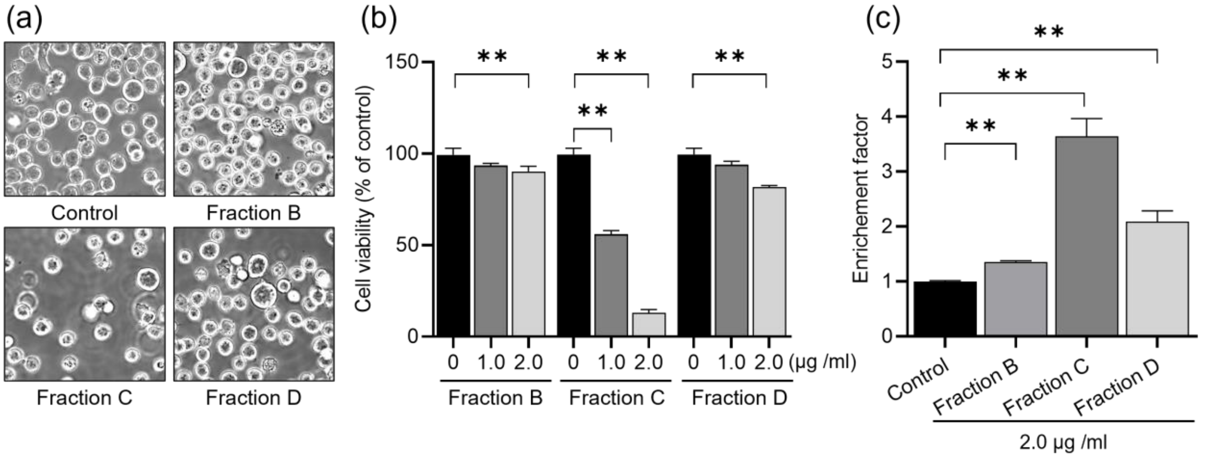

Figure S4. The effect of Cordyceps militaris-infused spirits on the proliferation inhibition and apoptotic induction of HL-60 cells. Morphological changes (a), proliferation inhibition effect (b) and DNA fragmentation induction (c) by treatment with Fractions B to D. The cells (5.0×10^3 cells/cm^2) were seeded in 96-well plates for 24 hours and then treated for 48 hours with indicated dose. The cell viability was measured by MTT assay. For DNA fragmentation assay, the cells (1.0×10^5 cells/cm^2) were seeded into 12-wells plates and then treated for 48 hours with 2.0 μg/ml of each Fractions. The DNA fragmentation was detected by Cell Death Detection ELISA^{PLUS} kit. Each value represents the mean ± S.D. of triplicate cultures and Columns with asterisk letters denoted significant differences (** $p < 0.01$).
